# Molecular Characterization of *Leptospira* Species Detected in the Kidneys of Slaughtered Livestock in Abattoirs in Gauteng Province, South Africa

**DOI:** 10.3390/pathogens12050666

**Published:** 2023-04-30

**Authors:** Banenat B. Dogonyaro, Henriette van Heerden, Andrew D. Potts, Folorunso O. Fasina, Arnau Casanovas-Massana, Francis B. Kolo, Christine Lötter, Charles Byaruhanga, Albert I. Ko, Elsio A. Wunder, Abiodun A. Adesiyun

**Affiliations:** 1 Department of Veterinary Tropical Diseases, Faculty of Veterinary Science, University of Pretoria, Onderstepoort 0110, South Africa; 2 Department of Epidemiology of Microbial Diseases, School of Public Health, Yale University, New Haven, CT 06520, USA; 3 National Veterinary Research Institute, Virology Department, Vom 930101, Nigeria; 4 Bacterial Serology Laboratory, ARC-Onderstepoort Veterinary Research, Onderstepoort 0110, South Africa; 5 ECTAD, Food and Agriculture Organization of the United Nations, Nairobi 00100, Kenya; 6 Gonçalo Moniz Research Center, Oswaldo Cruz Foundation, Brazilian Ministry of Health, Salvador 40081, Brazil; 7 Department of Production Animal Studies, Faculty of Veterinary Science, University of Pretoria, Onderstepoort 0110, South Africa; 8 Department of Paraclinical Sciences, School of Veterinary Medicine, The University of West Indies, St. Augustine 685509, Trinidad and Tobago

**Keywords:** isolation, molecular characterization, *Leptospira* spp., livestock, abattoirs, South Africa

## Abstract

*Leptospira* was investigated in kidneys (*n* = 305) from slaughtered livestock in the Gauteng Province abattoirs, South Africa, using a culture medium to isolate *Leptospira*, followed by the *LipL32* qPCR to detect *Leptospira* DNA. The *SecY* gene region was amplified, sequenced, and analyzed for *LipL32* qPCR-positive samples or *Leptospira* isolates. The overall frequency of isolation of *Leptospira* spp. was 3.9% (12/305), comprising 4.8% (9/186), 4.1% (3/74), and 0% (0/45) from cattle, pigs, and sheep, respectively (*p* > 0.05). However, with *LipL32* qPCR, the overall frequency of *Leptospira* DNA was 27.5%, consisting of 26.9%, 20.3%, and 42.2% for cattle, pigs, and sheep, respectively (*p* = 0.03). Based on 22 *SecY* sequences, the phylogenetic tree identified the *L. interrogans* cluster with serovar Icterohaemorrhagiae and the *L. borgpetersenii* cluster with serovar Hardjo bovis strain Lely 607. This study is the first molecular characterization of *Leptospira* spp. from livestock in South Africa. The reference laboratory uses an eight-serovar microscopic agglutination test panel for leptospirosis diagnosis, of which *L. borgpetersenii* serovar Hardjo bovis is not part. Our data show that pathogenic *L. interrogans* and *L. borgpetersenii* are circulating in the livestock population. Diagnostic use of molecular methods will eliminate or reduce the under-reporting of leptospirosis in livestock, particularly sheep, in South Africa.

## 1. Introduction

Pathogenic *Leptospira* spp. are the cause of leptospirosis in humans and animals worldwide. The disease is transmitted through exposure to the urine of an infected animal host or reservoir host containing the pathogenic leptospires. It can also be contracted from the environment [1]. A systemic infection due to the pathogen can affect an animal’s vital organs [2]. This disease could cause significant economic loss, especially to the livestock industry, and a threat to the human livelihood, as these livestock serve as a source of income and food [3,4].

Leptospirosis is caused by infection with the pathogenic *Leptospira* spp. [5]. In the past, all pathogenic strains were classified as *Leptospira interrogans,* and all non-pathogenic organisms (saprophytes) were placed under *Leptospira biflex* [6]. In recent times, *L. interrogans*, *L. borgpetersenii. L. alexanderi, L. alstonii, L. kirschneri, L. noguchi, L. santarosai, L. weilii,* and *L. wolffii* have been detected in clinical cases [7]. However, there has been an increase in the species of *Leptospira* resulting from the use of several molecular methods, including DNA–DNA hybridization, 16S rRNA analysis, multilocus sequence typing *(MLST),* and comparative genomics [Delgado et al. [8]. With the availability of inexpensive whole genome sequencing coupled with increased interest in metagenomics studies on environmental samples, the number of species of *Leptospira* has jumped from 22 in 2018 to 64 in 2019 [9].

The transmission of leptospirosis is attributed to many environmental factors [8,10]. This is through the excretion of leptospires in the urine of infected reservoir animals, where the pathogens are in close contact with domestic animals and rodents [1]. The pathogenesis of leptospirosis is not yet fully understood, but it has been reported that the pathogenic *Leptospira* spp. can result in different clinical manifestations in an infected host, ranging from subclinical infection to undifferentiated febrile illness [11,12]. The clinical signs of leptospirosis in animals include low milk production, abortion, stillbirth, infertility, decrease in meat production, and death [3,13]. The clinical signs and symptoms in humans include lethargy/depression, vomiting, fever, weight loss, polyuria/polydipsia, abdominal or lumbar pain, stiffness/arthralgia, renomegaly, diarrhea, icterus, oculonasal discharge, petechiae, weakness and dyspnea/cough [6]. Clinical signs and symptoms cannot confirm leptospirosis in animals and humans [1,7], but in animals, drop in milk production, abortions and reproductive failures. Since the signs may vary in other species, there is less concern on production-related symptoms. Therefore, the definitive diagnosis of the disease involves the use of specific and recommended diagnostic tools, such as bacteriological, serological (microscopic agglutination test, MAT), and molecular methods, which are considered mandatory in detecting the causative agent, pathogenic *Leptospira* spp. [4,14].

The type of samples processed for detecting *Leptospira* spp. is important [7]. Some diagnostic methods, such as bacteriological culture, are cumbersome, time-consuming, and easily contaminated, and require skilled personnel. More importantly, the isolation rate is frequently low and not sensitive [7]. These limitations pose a problem in obtaining data on leptospires circulating in animals, humans, and the environment in different regions. However, the advantage of the isolation method is that it is a standard technique for confirming infecting serovars from individual animals or humans [15,16].

The use of molecular diagnostic methods for leptospirosis is highly recommended [4] to reduce the problem of under-diagnosis of the disease. The methods include the qPCR detection of the pathogenic *Leptospira* spp. The major outer membrane *LipL32* partial gene region for screening [17] and the *secY* partial gene region, with its alternating conserved and variable regions, make it appropriate for heterogeneity interpretation of *Leptospira* spp. phylogeny [18]. In addition, the amplified *secY* partial gene region using the G1G2 internal primers [19], followed by sequence analysis, has allowed the identification of some serotypes or serovars [20,21], as well as the identification of pathogenic leptospires [18]. The advantages of the qPCR detection method, compared to the conventional methods, are that it is fast, it reduces the chances of contamination, it is specific and sensitive, especially with hydrolysis probes, and it has a high throughput [17,22]. The qPCR assay has been found to detect as low as 10^2^ and 10^3^ bacteria/mL of pure culture, whole blood, plasma, and serum samples targeting the *LipL32* and *SecY* gene regions [23]. Three independent experiments found a slightly higher sensitivity of qPCR in plasma than in whole blood and serum. However, the disadvantages of the qPCR method include its expensiveness, its requirement of good skills, and its inability to identify leptospires to serovars level [7].

Bacteriological isolation, serological assays, and PCR have been used singly or in combination to diagnose leptospirosis in animals and humans to increase the sensitivity and specificity of the diagnostic strategy [4,7]. The frequency of isolation of *Leptospira* spp. from the kidneys or urine of slaughtered livestock is variable: e.g., 0% in Brazil [24], 0.8% in Columbia [25], 10.4% in Zimbabwe [26], and 46.2% in another study in Brazil [27]. In a comparative study using three diagnostic methods, Rajeev et al. [28], in an abattoir survey of slaughtered cattle in Georgia, USA, reported a frequency of 78%, 29.7%, and 8.1% using fluorescent antibody staining, PCR, and culture, respectively. In Thailand, the use of both bacteriological assay and PCR revealed an overall frequency of detection of *Leptospira* of 12.21% (16/131), compared with an isolation rate of 0.76% (1/131) [29]. This variation could be due to the higher sensitivity of molecular methods, such as the qPCR, than conventional methods for isolating *Leptospira* spp. Furthermore, several studies have documented that the frequency of *Leptospira* DNA in kidney tissues is consistently higher than the rate of isolation of the organism from the same kidney tissues [24,28,29].

In South Africa, the last reported isolation of leptospires was documented in 1987 [30], where 25% (3/12) of bovine tissues cultured for *Leptospira* spp. were positive, but the isolates were not serotyped. Over the past two decades, no studies on livestock on farms or at abattoirs investigated the prevalence of leptospirosis. Therefore, the objectives of this study were to determine the frequency of isolation of *Leptospira* spp. from the kidneys of slaughtered cattle, pigs, and sheep at abattoirs in Gauteng Province, South Africa, and to detect pathogenic *Leptospira* spp. using the *Leptospira* qPCR assay targeting the *LipL32* gene region from which the pathogenic *Leptospira* spp. were characterized using *SecY* partial gene region sequences in the kidneys collected from slaughtered livestock and abattoir effluents.

## 2. Materials and Methods

### 2.1. Study Area

The study was conducted in South Africa, located in the southern tip region of Africa, with a population of approximately 57.78 million people as of 2018. Gauteng Province, the study area, is in the Highveld and is the smallest province in South Africa, accounting for only 1.5% of the land area (18.178 km^2^) of the country’s total area of 1220.813 km^2^. The province has the highest number of abattoirs in the country, comprising high throughput (HT) and low throughput (LT) abattoirs that slaughter animals from Gauteng Province and other provinces. Therefore, the slaughtered animals sampled at the abattoirs in Gauteng Province in the current study may be representative of slaughtered animals throughout the country, as they originated from provinces across South Africa. The population of livestock per million in 2014/2015 in Gauteng Province was reported to be 13.7, 11, and 1.5 for cattle, sheep, and pigs, respectively [31], and included the livestock species sampled in the current study.

### 2.2. Location of Abattoirs

**Figure 1:**
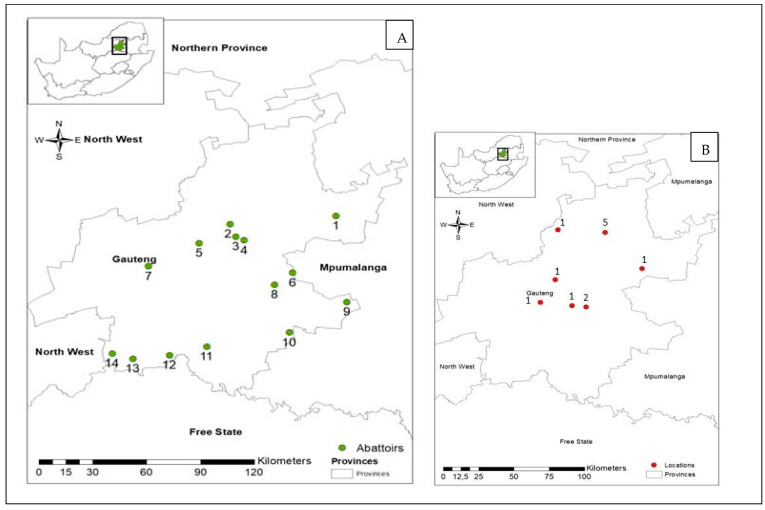
(**A**) The map shows the location of Gauteng Province in South Africa, and the main map displays the locations of the 14 abattoirs in Gauteng Province from which samples were collected. (**B**) The distribution of livestock that were positive for *Leptospira* spp. by isolation in abattoirs in the Gauteng Province shows the number of *Leptospira* spp. recovered by abattoirs.

A list of functional red meat abattoirs (mono- and multi-species) in Gauteng Province was provided by Gauteng Department of Agriculture and Rural Development (GDARD). Overall, 14 abattoirs comprising seven HT and seven LT were randomly selected from abattoirs whose owners approved the conduct of the study at their facilities. The distribution of abattoirs in Gauteng Province from which livestock were sampled and of livestock that were positive for *Leptospira* spp. by isolation is shown in Figure 1A,B. Geographic information system (GIS) data were collected using the Garmin Nüvi^®^ GPS navigator (Garmin Ltd., Lenexa, KS, USA.). The readings were entered into the Arc GIS program version 13.0 (Environmental Systems Research Institute, Redlands, CA, USA), and the data were used to plot figures and produce maps.

### 2.3. Type of Study and Sampling

This cross-sectional study consisted of convenient sampling at 14 red-meat abattoirs from slaughtered livestock in Gauteng Province in South Africa, sampled between September 2016 and April 2017. The animals slaughtered at Gauteng abattoirs were not exclusively from farms in the province, as this province allows movement from other provinces. The abattoir owners or managers provided consented to facilitate the study.

### 2.4. Demographic Data and Risk Factors for Livestock Sampled at the Abattoirs

The demographic data obtained from the abattoirs included the type of abattoir (HT or LT) and the location of abattoirs using the Global Positing System (GPS) within Gauteng Province. Animal-level risk factors were obtained to investigate their potential effects on the detection frequency of *Leptospira* spp. and included the animal species (cattle, pigs, and sheep), sex (male and female), age (adult and young), and breed.

### 2.5. Samples Collected

The animals sampled in this cross-sectional study were cattle, pigs, and sheep. The kidneys of slaughtered livestock (*n* = 305) were collected (one kidney per animal) from 186, 74, 45, and 14 cattle, pigs, sheep, and abattoir effluents, respectively. The kidney samples were aseptically removed from each randomly selected carcass into individual sterile Ziploc bags. Abattoir effluent samples were also aseptically collected in three different locations at random within the abattoirs, and each abattoir sample was pooled in a 50 mL plastic cup and labeled. All collected samples were transported on ice to the laboratory within 2–4 h of collection. Each kidney sample from the 305 animals was processed using both bacteriological culture (isolation of *Leptospira* spp.) assay and molecular methods (detection of *Leptospira* DNA) assay. Pooled abattoir effluent samples from each of the abattoirs (*n* = 14) were centrifuged into pellets for DNA extraction.

### 2.6. Isolation of Leptospira *spp.*

Ellinghausen–McCullough–Johnson–Harris (EMJH) semi-solid medium (Difco™ BD *Leptospira* Enrichment EMJH, USA) was prepared by adding 1% agar to the basal broth media, and EMJH liquid medium was used for the purification of leptospiral cultures for further typing and characterization. The isolation of *Leptospira* spp. from the kidney samples (*n* = 305) was conducted at the Agricultural Research Centre-Onderstepoort Veterinary Research laboratory, Onderstepoort Gauteng Province, South Africa. Kidney tissues (50 mg) containing the cortex and medulla portion were aseptically cut using sterile scalpel blades in a class II biohazard cabinet (BSL 2) and added to a sterile 5 mL syringe plunger containing 3 mL of liquid EMJH medium to macerate the tissues. Approximately 2 mL of the macerated kidney contents were transferred aseptically for homogenization using the Precellys^®^ 24 lysis homogenizer at 4500 rpm for 2 min. After that, 200 µL of the supernatant was aseptically inoculated into 5 mL of semi-solid EMJH medium [32] containing 200 μg/mL 5-fluorouracil in a labeled 10 mL sterile tube. The inoculated EMJH media tubes were incubated at 29 °C and observed weekly for 3–6 months using dark-field microscopy (Nikon Labophot^®^ Japan; model number: 277602) for the presence or absence of leptospires. Samples without leptospiral growth by the end of the six-month incubation were classified as negative for *Leptospira* spp. (Supplementary data: Figure S1). Isolates positive for *Leptospira* spp. were identified under the dark-field microscope.

### 2.7. Detection of Leptospira *spp.* by Real-Time PCR (qPCR) Using the Pathogenic LipL32 Gene Region

The pelleted abattoir effluents were resuspended with 2 mL PBS. DNA was extracted using the ISOLATE II Genomic DNA (Bioline) kit as described by the manufacturers, with minor modifications—specifically, the use of 50 mg of tissue and 2 h of incubation instead of 25 mg of tissue and 3 h of incubation of the sample, with the addition of pre-lysis buffer and proteinase K. These extractions were carried out at the Department of Veterinary Tropical Diseases Laboratory, Faculty of Veterinary Science, University of Pretoria, South Africa.

Extracted DNA from the *Leptospira* spp. isolates and the kidney tissues were tested at the Yale University School of Public Health, USA, using the TaqMan Cador^®^*Leptospira* qPCR commercial kit on the Rotor Gene^®^ Q (Whitehead Scientific, Germany^®^) to hybridize with the *LipL32* gene region of pathogenic *Leptospira* spp., following the manufacturer’s instructions.

A *LipL32* qPCR assay, as described by Wunder et al. [17], was conducted at the Yale University School of Public Health, USA, using a standard stock positive control genomic DNA (*Leptospira interrogans* serovar Copenhageni strain Fiocruz L1-130 isolated by Nascimento et al. [33]. A standard curve calibration of the genomic DNA was constructed using the serial dilution of positive control DNA starting at Log10^1^ to Log10^7^ genomic equivalents per gram (GEq/mL). The extracted DNA kidney and abattoir effluent samples were tested in duplicate alongside each standard curve dilution. A non-template negative control was also tested with all samples, using the genomic equivalents per mg of kidney DNA to express the results [34]. After the standardization of the standard curve, the *Leptospira* DNA extracts were subjected to *LipL32* gene qPCR targeting to screen for pathogenic *Leptospira* spp. [17]. The PCR assays consisted of a 25 µL final volume containing 1x Platinum Quantitative PCR Supermix Rox-UDG (Invitrogen^®^), 10 µM of each primer (LipL32-45F and LipL32-286R), 5 µM TaqMan probe (LipL32-189P), and 5 µL of extracted DNA. The cycling conditions were as previously described, with a holding stage of 95 °C for 10 min, 45 cycles of 95 °C for 15 s, and 60 °C for 1 min using a TaqMan-based quantitative PCR assay in ABI 7500 system (Thermo Fisher Scientific, real-time PCR ABI 7500). The Ct-value ≤ 40 was regarded as positive, while a Ct-value ≥ 40 was regarded as negative. Excel software determined the standard curve correlation efficiency (R^2^). The *LipL32* commercial qPCR and the *LipL32* qPCR described by Wunder et al. [17] have the same target and are reported as *LipL32* qPCR results.

### 2.8. Detection and Characterization of Leptospira *spp.* SecY Gene Region PCR and Sequencing

Ten pure isolates of *Leptospira* spp. were subjected to the pathogenic *SecY* gene PCR [18]. The pathogenic *SecY* PCR assay to discriminate the pathogenic *Leptospira* spp. [18] was amplified from *LipL32* qPCR positive kidney DNA with concentrations over Log10gc/g 4.23, as determined with a standard curve (Supplementary Figure S2) followed by sequencing. The pathogenic *SecY* partial gene region was amplified using the SecYII and SecYIV primer sets: SecYII (5′-GAATTTCTCTTTTGATCTTCG-3′) and Sec IV (5′-GAATTTCTCTTTTGATCTTCG-3′) for the first step PCR with a final volume of 25 µL containing 1× buffer, 200 µM dNTPs, 400 µM primer pair SecYII and SecYIV each, 0.2 µg bovine serum albumin (BSA) (Ambion), 1.25U Taq polymerase, (Thermo Scientific), and 3 µL extracted DNA template [16]. The nested PCR was performed using the G1G2 pair of primer sets for pathogenic *Leptospira* spp., with a total volume of 25 µL containing 1x buffer, 200 µM dNTPs, 400 µM of each primer SecYII and SecYIV, 0.2 µg bovine serum albumin (BSA), (Ambion), 1.25 U Taq polymerase (Thermo Scientific), and 3 µL of the first PCR amplicon [18]. The PCR reactions for the first and nested *SecY* partial gene amplification consisted of 94 °C for 5 min (1 cycle), 94 °C for 30 s, 55 °C for 45 s (35 cycles), and 72 °C for 60 s (1 cycle) in a My Cycler™ Thermal Cycler (BioRad). The positive control used for the amplification was *L. interrogans,* serovar Copenhageni strain Fiocruz L1-130 [33], and ultra-pure water (Thermo Scientific) was used as the negative control. The agarose gel electrophoresis was run using 3 µL of amplicons in 1.5% agarose gels in TBE buffer for 35 min at 110 V, using ethidium bromide (10 mg/µL). Images were captured using the Bio-Rad-Chemi-Doc-XRS. The Qiaquick PCR purification kit was used per the manufacturer’s specifications to purify the nested *SecY* PCR products (285 bp). The generated amplicons were then sent to Eurofins Genomic (Bayern, Germany), for Sanger sequencing.

### 2.9. Sequence Analyses of SecY Partial Gene Region of Leptospira Isolates and Kidney Tissue Samples and Phylogeny

The resulting sequences obtained from PCR products obtained from *Leptospira* spp. isolates and kidney samples were edited using the CLC Genomics Workbench, version 7.5.1. Reference sequences were blasted using the basic local alignment search tool (BLAST) (http://www.ncbi.nih.gov, accessed on 15 March 2020). The sequenced *SecY Leptospira* and *Leptospira* reference sequences retrieved from GenBank were aligned using MAFFT version 7 (https://mafft.cbrc.jp/alignment/server/, accessed on 24 March 2020) and trimmed using the BioEdit (http://www.mbio.ncsu.edu/BioEdit/page2.html, accessed on 14 May 2020). A phylogenetic tree was constructed using the maximum likelihood method in MEGA 7.0.2 with a 1000 bootstraps value.

### 2.10. Statistical Analyses of Risk Factors

Univariate analysis of associations was conducted using the isolation frequency of the animal as a binary outcome (positive or negative). The predictor variables were the abattoir (14 abattoirs), the type of abattoir (multi-species and mono-species), the throughput of the abattoir (LT and HT), the animal species (cattle, sheep, and pigs), sex (male, female), and age (adult and young). Each predictor variable was tested for significant associations with the serological status using the chi-square test of association. The prevalence ratio for each animal-level potential risk factor was obtained and 95% confidence intervals were estimated using the quantiles formation of the normal distribution (qnorm) with the MASS package in R [35].

Significant variables (*p* < 0.05) in the univariate analysis were assessed for collinearity using the chi-square statistic; variables were considered collinear if *p* < 0.05. When a pair of variables was found to be collinear, only the more biologically plausible variable was kept for further analysis in the binary logistic regression. The analysis considered the isolation frequency as determined by the isolation of *Leptospira* spp. and the detection of *Leptospira* DNA by PCR for individual animals as a binary outcome. Of the three statistically significant variables (abattoir, breed, species) from the univariate analysis, the pairs breed and species, abattoir and breed, and abattoir and species were found to be collinear and, therefore, only species and abattoir were retained in the final model.

Given the likelihood that some animals slaughtered in the same abattoir may have originated from the same farm/herd/flock, leading to dependence, intra-cluster correlation within abattoirs was tested at the beginning of the regression process. To test if seropositivity for antibodies to *Leptospira* spp. by the MAT were clustered in abattoirs, a log ratio test between a model with the “abattoir” as a random effect and a null model was performed. The *p*-value from the log ratio test was less than 0.05, meaning that the results of *Leptospira* spp. were clustered inside the abattoir.

A mixed-effect logistic regression model was used in the multivariable analysis, with the species as the “fixed effect” and the abattoir as the “random effect.” Hosmer–Lemeshow χ^2^ was used as a goodness-of-fit test. Statistical analysis was performed using R Console version 3.2.1 [36] at a 5% significance level. For the cleaning of data and frequency determination of the predictor variables of the livestock slaughtered, Microsoft Excel 2010 was used for descriptive statistics to plot the bar chart and to determine the frequency of all the variables used, as mentioned in the risk factors analyses.

### 2.11. Ethical Approvals

Animal ethics approvals were obtained from the animal ethics committee of the University of Pretoria, Faculty of Veterinary Science (Number: v084-1 the ARC-OVR (Number: AEC12-16). Section 20 approval from the Department of Agriculture, Land Reform and Development (DALRD), was also obtained.

## 3. Results

### 3.1. Isolation of Leptospires from Livestock Kidneys by Isolation and Risk Factors

The overall frequency for the isolation of leptospires from slaughtered livestock kidneys in 14 Gauteng abattoirs was 3.9% (12/305). The Dingers ring zone was observed 3 to 8 weeks post-inoculation in EMJH media inoculated with kidney samples with leptospiral growth (Supplementary data: Figure S1). Of the 12 isolates identified as possible *Leptospira* spp. from kidney tissues using dark-field microscopy, four isolates tested negative for the pathogenetic *LipL32* qPCR. Two of the remaining eight *Leptospira* spp. isolates were contaminated, resulting in six pure pathogenic *Leptospira* spp. isolates.

For the throughput of abattoirs, the frequency of isolation of *Leptospira* spp. was 50% (6/12) and 50% (6/12) from HT and LT abattoirs, respectively. The frequency of isolation by animal species was 4.8% (9/186), 4.1% (3/74), and 0.0% (0/45) in cattle, pigs, and sheep, respectively, but the differences were not statistically significant *(p* > 0.05). Of the 12 *Leptospira* spp., nine (75%), three (25%), and 0 (0%) originated from cattle, pigs, and sheep, respectively. For cattle, the frequency of isolation of *Leptospira* spp. was 5.3% (9/170) and 0% (0/16) for adult and young animals, respectively, but the difference was not statistically significant (*p* > 0.05). For pigs, the frequency of isolation of *Leptospira* spp. was 2.3% (1/43) and 6.5% (2/31) for adult and young animals, respectively (*p* > 0.05).

For cattle, the overall frequency of isolation of *Leptospira* spp. was 1.8% (2/110) and 9.2% (7/76) for male and female cattle, respectively (*p* = 0.0209). Thus, of the nine isolates recovered from cattle, the majority, 77.8% (7/9), were from females and the minority, 22.2% (2/9) from males. For pigs, the frequency of isolation of *Leptospira* spp. was 2.0% (1/50) and 8.3% (2/24) for male and female pigs, respectively *p* > 0.05). Of the three isolates recovered from pigs, 33.3% (1/3) were males and 66.7% (2/3) were females.

For cattle, the frequency of isolation of *Leptospira* spp. was 13.3% (4/30) and 3.9% (5/129) from Nguni and Bonsmara cattle, respectively *(p* = 0.28), with 55.6% (5/9) of the isolates from the Nguni breed and 44.4% (4/9) from the Bonsmara breed. For pigs, the frequency of isolation of *Leptospira* spp. was 4.1% (3/74), but all the pigs slaughtered and sampled were of the large white breed.

### 3.2. Detection of Leptospira *spp.* in Kidneys of Livestock and Abattoir Effluents by qPCR and Risk Factors

The overall frequency of pathogenic *Leptospira* spp. detected with *LipL32 gene* qPCR in kidney tissues of livestock (cattle, pigs, and sheep) was 27.5% (84/305) for the kidney tissues samples analyzed, but all 14 abattoir effluent samples were negative. Supplementary data: Figure S2 show the standardized qPCR curve used to quantify the concentrations of a standard stock positive control genomic DNA (*Leptospira interrogans*, serovar Copengageni strain Fiocruz L1-130). The frequency pathogenic *Leptospira* spp. detected with *LipL32 gene* qPCR in cattle kidney tissues was 6.9% (50/186) (Supplementary data: Figure S3). The frequency of pathogenic *Leptospira* spp. detected with *LipL32* gene qPCR of pigs was found to be 20.3% (15/74) (Supplementary data: Figure S4).

Of the three animal species tested, sheep had the highest frequency of pathogenic *Leptospira* spp. Detected, with *LipL32* gene qPCR at 42.2% (19/45) (Supplementary data: Figure S5). The isolation rate of *Leptospira* spp. was significantly lower than the detection rate for *Leptospira* DNA in kidney tissues in cattle (4.8% versus 26.9%, *p* < 0.0001), pigs (4.1% versus 20.3%, *p* = 0.0025), and sheep (0% versus 42.2%, *p* < 0.001).

**Figure 2:**
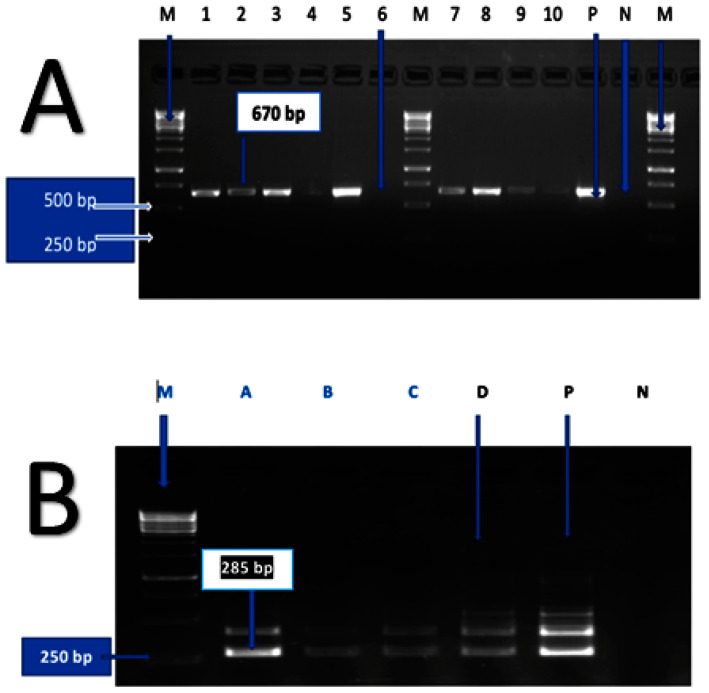
Agarose gel images. (**A**) First amplification of the 670 bp *secY* partial gene region using PCR with primers (SecYII and SecYIV). The marker (M) is the O’ Gene Ruler 1Kb DNA Ladder (Thermo Fischer). M = Marker; 1, 2, 3,4, 5, 7, 8, 9, and 10 = samples positive; 6 = sample negative; P = positive control (*Leptospira interrogans,* serovar Copenhageni strain Fiocruz L1-130) and N = negative control (ultra-pure water). (**B**) Nested amplification of the 285 bp *SecY* partial gene region using PCR with primers (G1G2). The O’ Gene Ruler 1Kb DNA Ladder (Thermo Fischer) was used as a marker (M). M = marker; A to D = samples positive for *SecY* gene region nested PCR; P = positive control (*Leptospira interrogans,* serovar Copenhageni strain Fiocruz L1-130) and N = negative control (ultra-pure water).

The overall detection of the *LipL32* gene region using qPCR present in pathogenic *Leptospira* spp. was positive in 84 (27.5%) of the 305 kidney samples tested. The positivity rate was 3.3% (10/305) for *Leptospira* isolates observed under the dark-field microscope (only 10 of the 12 *Leptospira* isolates were regarded as pure, and two were contaminated and could not be used in this assay). Of the 10 isolates of *Leptospira* spp. observed under the dark-field microscope, six were identified as pathogenic *Leptospira* spp. by the *LipL32* gene region qPCR assay and the remaining four isolates were unidentified. Furthermore, from the positive genomic DNA quantified, the *SecY* gene region of *Leptospira* spp. generated 22 sequences from the 285 bp *SecY* partial gene region, consisting of the six *Leptospira* isolates and 16 from the kidney tissues. Figure 2 shows the amplification of the first and nested PCR of the *SecY* partial gene region using PCR.

### 3.3. Phylogeny of SecY Sequences of Leptospira Isolates and Kidneys Samples Tissue

As indicated, 22 sequences from the 285 bp amplified *SecY* gene region were aligned, comprising sequences from six isolates, and 16 from kidney tissues were identified. The *SecY* gene region sequences from the six isolates included five from cattle (four *L. interrogans* and one *L. borgpetersenii*) and one *L. interrogans* from a pig. The *SecY* gene region sequences from the 16 kidney tissue samples included 10 from cattle, of which nine were *L. interrogans,* and one was *L. borgpetersenii.* Three *SecY* gene region sequences from pig kidney samples were identified as *L. interrogans,* and the three sequences from sheep were identified as two *L. interrogans* and one *L. borgpetersenii.*

**Figure 3:**
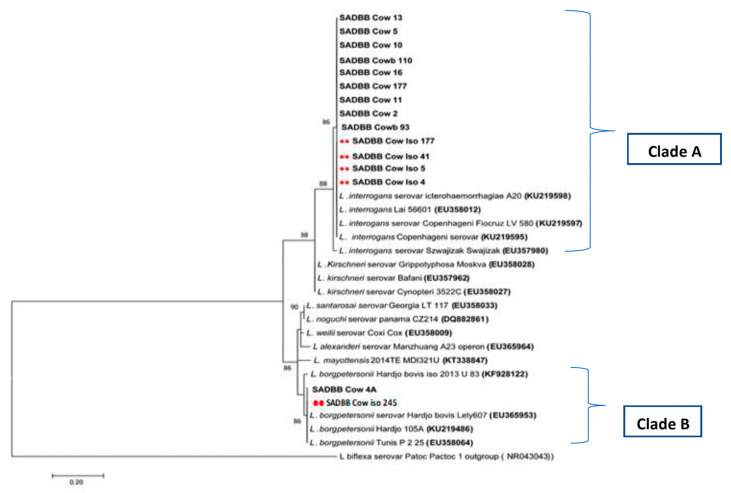
Phylogenetic tree of *SecY* partial gene region of pathogenic *Leptospira* spp. sequences using the maximum likelihood methods based on the General Time Reversible (GTR+1) model. *SecY* sequences were obtained from 15 cattle slaughtered at Gauteng abattoirs, indicated in bold, including sequences obtained from *Leptospira* cultures or isolates indicated by red dots and GenBank reference sequences pathogenic *Leptospira* species with *L. bifexa* as an outgroup. The *SecY Leptospira* gene sequences from cattle clustered into two clades namely clades A consisting of *L. interrogans* and clade B consisting of *L. borgpetersenii* sequences. In clade A, *SecY* sequences of four isolates from cows and nine from kidney samples were identical to each other and to GenBank sequences of *L. interrogans* serovar Icterrohaemorrhagiae A 20 (KU219597), *L. interrogans* Lai 56,601, and *L. interrogans* serovar Copenhageni. In clade B, two *SecY L. borgpetersenii* sequences from cattle samples were identical to each other and Genbank *L. borgpetersenii* serovar Hardjo bovis strain Lely 607 (EU365953), *L. borgpetersenii* serovar Hardjo 105A, and *L. borgpetersenii* Tunis P 2 25 sequences. A bootstrap of 1000 replicates with values above 75% was considered.

The phylogenetic tree analysis of *SecY Leptospira* gene sequences from cattle of *L. interrogans* and *L. borgpetersenii* clustered into two clades (clades A and B) according to their serovars (Figure 3). The four *SecY L. interrogans* sequences (four from isolates indicated by red dots) and nine from kidney samples (in bold without dots) (Figure 3, clade A) were identical to each other and to GenBank sequences of *L. interrogans* serovar Icterrohaemorrhagiae A 20 (KU219597), *L. interrogans* Lai 56,601, and *L. interrogans* serovar Copenhageni (Figure 3, clade A) and clustered with nine identical sequences from kidney samples (in bold without dots) (Figure 3, clade A). The two *SecY L. borgpetersenii* sequences from cattle samples (one isolate with the red dot and one kidney tissue sample in bold without the dot) were identical to each other and Genbank *L. borgpetersenii* serovar Hardjo bovis strain Lely 607 (EU365953), *L. borgpetersenii* serovar Hardjo 105A, and *L. borgpetersenii* Tunis P 2 25 sequences (Figure 3, clade B).

**Figure 4:**
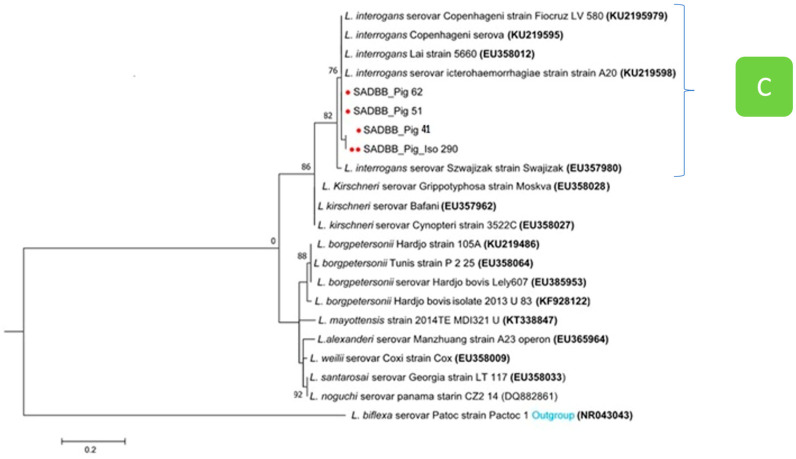
Phylogenetic tree of *SecY* partial gene region of pathogenic *Leptospira* spp. sequences using the maximum likelihood methods based on the General Time Reversible (GTR+1) model. *SecY* sequences were obtained from four pigs slaughtered at Gauteng abattoirs, indicated by a bold single dot (kidney tissues), including sequences obtained from *Leptospira* cultures or isolate, indicated by two red dots, GenBank reference sequences of pathogenic *Leptospira* species with *L. bifexa* as an outgroup. In clade C, sequences *SecY* SADBB_pig_62 and SADBB_pig_51 from pig kidney samples were identical with *L. interrogans* serovar Icterrohaemorrhagiae A 20 (KU219597), *L. interrogans* Lai 56,601, and *L. interrogans* serovar Copenhageni, while isolate SADBB_pig_iso 290 sequence and SADBB_pig_41 from pig kidney sample were identical to each other. A bootstrap of 1000 replicates with values above 75% was considered.

The phylogenetic tree analysis of *secY Leptospira* gene sequences from pigs identified as *L. interrogans* clustered into clade C (Figure 4). Sequences *SecY* SADBB_pig_62 and SADBB_pig_51 from pig kidney samples were identical with *L. interrogans* serovar Icterrohaemorrhagiae A 20 (KU219597), *L. interrogans* Lai 56,601, and *L. interrogans* serovar Copenhageni, while *SecY L. interrogans* sequences from an isolate (SADBB_pig_iso 290, indicated by two red dots) and SADBB_pig_41 (from pig kidney sample) were identical to each other but differed slightly from different *L. interrogans* sequences (Figure 4, clade C).

**Figure 5:**
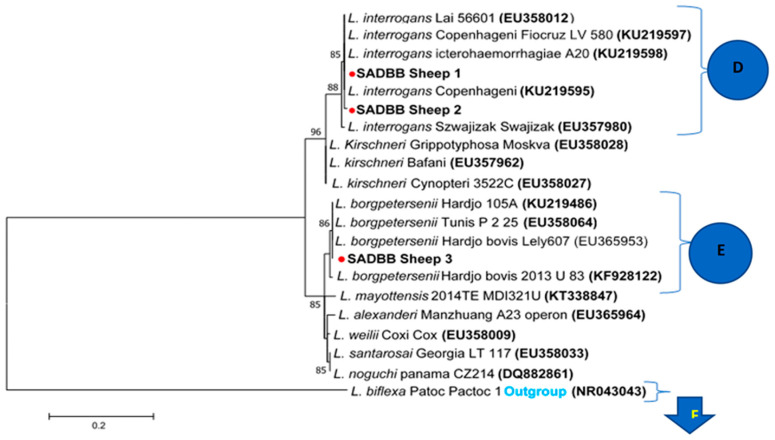
Phylogenetic tree of *SecY* partial gene region of pathogenic *Leptospira* spp. sequences using the maximum likelihood methods based on the General Time Reversible (GTR+1) model. *SecY* sequences were obtained from 3 slaughtered sheep at Gauteng abattoirs, indicated with one red dot, as GenBank reference sequences of pathogenic *Leptospira* species with *L. bifexa* as an outgroup. The gene sequences from sheep clustered with *L. interrogans* (clade D) and *L. borgpetersenii* (clade E). In clade D, *SecY L. interrogans* SADBB sheep 1 sequence from a kidney sample was identical to GenBank sequences of *L. interrogans* serovar Icterohaemorrhagiae A 20 (KU219597), *L. interrogans* Lai 56,601, and *L. interrogans* serovar Copenhageni and differed slightly from SADBB Sheep 2 sequence from sheep kidney. Clade E, the SADBB sheep 3 sequence from sheep kidney was identical to Genbank *L. borgpetersenii* serovar Hardjo bovis strain Lely 607 (EU365953), *L. borgpetersenii* serovar Hardjo 105A, and *L. borgpetersenii* Tunis P 225 sequences. A bootstrap of 1000 replicates with values above 75% was considered.

The phylogenetic tree analysis of *SecY Leptospira* gene sequences from sheep consisting of *L. interrogans* and *L. borgpetersenii* clustered into two clades (clades D and E) according to the different serovars (Figure 5). The *SecY L. interrogans* SADBB sheep one sequence from a kidney sample (in bold with one red dot, Figure 5**,** clade D) was identical to GenBank sequences of *L. interrogans* serovar Icterohaemorrhagiae A 20 (KU219597), *L. interrogans* Lai 56,601, and *L. interrogans* serovar Copenhagen (Figure 5, clade D), which clustered but differed slightly from *SecY L. interrogans* SADBB sheep 2 sequences from a kidney sample (in bold with one red dot, Figure 5, clade D). The *SecY L. borgpetersenii* SADBB sheep 3 sequences (in bold with one red dot) from the sheep kidney sample was identical to Genbank *L. borgpetersenii* serovar *Hardjo bovis* strain Lely 607 (EU365953), *L. borgpetersenii* serovar Hardjo 105A, and *L. borgpetersenii* Tunis P 225 sequences (Figure 5, clade E).

**Figure 6:**
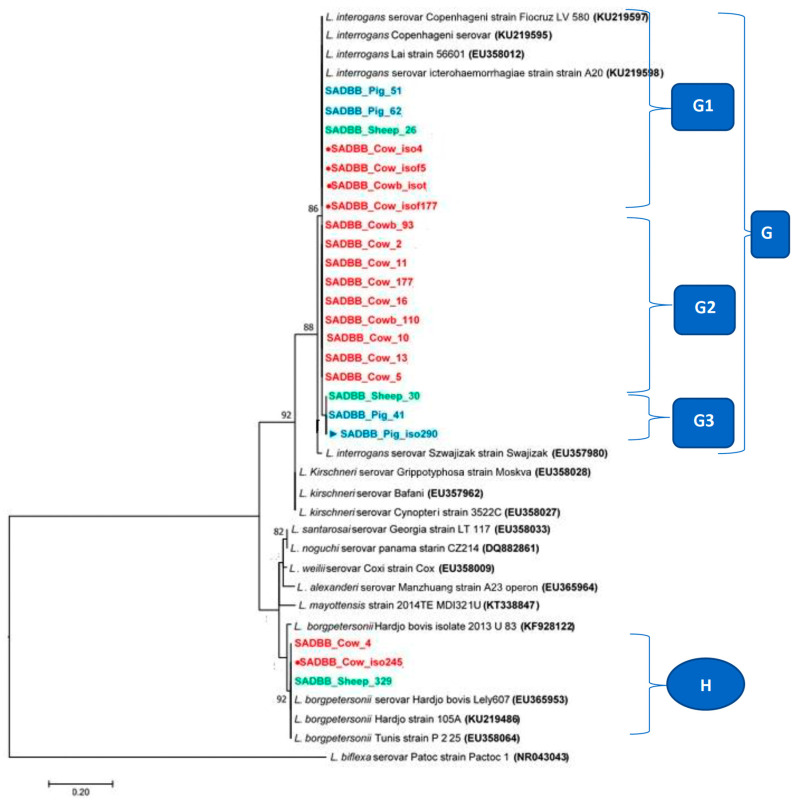
Phylogenetic tree of *SecY* partial gene region of pathogenic *Leptospira* spp. sequences using the maximum likelihood methods based on the general time reversible (GTR+1) model. *SecY* sequences were obtained from 22 livestock [cattle (red ink in bold), pigs (blue ink in bold), and sheep (green ink in bold)] slaughtered at Gauteng abattoirs. Cattle isolates sequences are indicated with red dots and red ink in bold, while cattle kidney tissue samples sequences are indicated by red ink in bold without a dot; pigs isolates are indicated by a blue arrow and written in blue ink in bold, while the pigs’ kidney tissue samples sequences are marked with blue written in bold ink without the arrow and the sheep kidney tissues sequences are written in bold green ink. The G1 clade consisted of sequences from isolates from cows’ kidneys SADBB_cow_iso4, SADBB_cow_isof5, SADBB_cowb_isot, and SADBB_cow_isof177, pigs’ kidney tissues samples, SADBB_Pig_62 and SADBB_Pig_51, and the sheep kidney sample, SADBB_sheep_26 that were identical to *L. interrogans* serovar icterohaemorrhagiae strain A20 (KU219598), *L. interrogans* Lai strain 56,601 (EU358012), *L. interrogans* Copenhageni serovar (KU219595), and *L. interrogates* serovar Copenhageni strain Fiocruz LV 580 (KU219597). In G2 clade, nine sequences from cattle kidney tissue were identical. The *SecY L. interrogans* sequences from pig culture (SADBB_Pig_iso290, SADBB_pig_41 and SADBB_Sheep_30) were identical and clustered together in subclade G3. In clade H, the *SecY L. borgpetersenii* SADBB_Cow_4, SADBB_Cow_iso245, and SADBB_Sheep_329 sequences were identical with Genbank L. *borgpetersonii* serovar Hardjo bovis Lely607 (EU365953), *L. borgpetersonii* Hardjo strain 105A (KU219486), and *L. borgpetersenii* Tunis strain P 225 (EU 358064) sequences.The GenBank reference sequences of pathogenic *Leptospira* species with *L. bifexa* as an outgroup were used. A bootstrap of 1000 replicates with values above 75% was considered.

The phylogenetic tree analysis of *Leptospira* spp. *SecY* partial gene region sequences from cattle, pigs, and sheep clustered with Genbank partial *SecY* sequences of *L. interrogans* and *L. borgpetersenii* into two clades (G and H) (Figure 6). The *SecY* partial gene sequences from isolates recovered from cows’ kidneys SADBB_cow_iso4, SADBB_cow_isof5, SADBB_cowb_isot, and SADBB_cow_isof177 (Figure 6, G1 marked with red dots written in red ink boldly), pigs’ kidney tissues samples, SADBB_Pig_62 and SADBB_Pig_51, (G1 in blue ink in Figure 6), and the sheep kidney sample, SADBB_sheep_26 (G1 in green ink in Figure 6) were identical to *L. interrogans* serovar icterohaemorrhagiae strain A20 (KU219598), *L. interrogans* Lai strain 56,601 (EU358012), *L. interrogans* Copenhageni serovar (KU219595), and *L. interrogates* serovar Copenhageni strain Fiocruz LV 580 (KU219597) (Figure 6, G1). These identical *L. interrogans* South African sequences (G1) were from pig and sheep kidney tissues and isolates from cattle kidneys. Nine *SecY* partial sequences from cattle kidney tissue were identical (G2 subclade in Figure 6) and slightly different from sequences in the G1 subclade. The *SecY L. interrogans* sequences from pig culture (SADBB_Pig_iso290, indicated by the blue arrow and written in blue ink in bold), SADBB_pig_41 and SADBB_Sheep_30 (from pig kidney tissue, blue ink in bold), and sheep kidney tissue (green ink in bold) samples) were identical and clustered together in subclade G3 and differed slightly from G2 subclade (Figure 6). The *SecY L. borgpetersenii* SADBB_Cow_4, SADBB_Cow_iso245, and SADBB_Sheep_329 sequences (Figure 6, clade H) were identical with Genbank L. *borgpetersonii* serovar Hardjo bovis Lely607 (EU365953), *L. borgpetersonii* Hardjo strain 105A (KU219486), and *L. borgpetersenii* Tunis strain P 225 (EU 358064) sequences.

## 4. Discussion

Accurate diagnosis of leptospirosis in livestock is important for the wellbeing of animals, the economy of the country, small stakeholders’ livelihoods, and a healthy environment. Such accuracy is also invaluable for preventing and controlling zoonotic disease spillover to humans, especially veterinarians, abattoir workers, and farmers [1,3,4]. Thus, this work is of vital importance. The overall frequency of isolation of *Leptospira* spp. (3.9%) from all livestock in our study (cattle, pigs, and sheep) is considerably lower than that reported for slaughtered livestock elsewhere; for instance, the frequency in a Brazilian slaughterhouse was 38.2% (13/34) and in Harare, Zimbabwe, it was 10.4% [26,27]. It was, however, higher than the figure obtained from Nan province, Thailand (0.76%; 1/131) [29]. The difference may be reflective of livestock management and sanitary practices, among other factors [30]. Of diagnostic relevance is the finding that the isolation rate of *Leptospira* spp. was significantly lower than the detection rate for *Leptospira* DNA in kidney tissues of cattle (4.8% versus 26.9%, *p* < 0.0001), pigs (4.1% versus 20.3%, *p* = 0.0025) and sheep (0% versus 42.2%, *p* < 0.000001). Furthermore, the overall frequency of isolation of *Leptospira* spp. from kidney tissues of all livestock tested was 3.9% (12/305), compared with a detection rate of 27.5% (84/305) of *Leptospira* DNA in kidney tissues (*p* < 0.00001). Similar patterns have been documented in other studies [24,29]. The implication is that the DNA detection outperformed the isolation procedure; focusing on the latter alone to determine the prevalence of *Leptospira* spp. in livestock will grossly underestimate the true disease prevalence. Significantly, though, all the sheep kidneys were negative for *Leptospira* spp. by culture but were positive for *Leptospira* DNA. Hence, the qPCR assay’s sensitivity, specificity, and accuracy are considerably higher than those of any other diagnostic methods used to diagnose leptospirosis [7,14,37]. However, a qPCR assay cannot differentiate between the existence of live and dead leptospires in kidney tissues, which limits its application for risk assessment for human and animal exposure to pathogen infection and environmental contamination with viable leptospires [7,17]. Freitas et al. [24] reported different isolation rates using different samples, with urine and liver samples outperforming uterus, kidney, and ovarian tissues [38]. Hence, a suitable sample must be combined with the test method to optimize the isolation and characterization of the pathogen.

Abattoir workers have a higher prevalence (serology and isolation) of leptospirosis and other zoonoses than the general population [29]. Human exposure to *Leptospira* spp. can be through the mucous membranes and skin [1]; this has been documented in South Africa, mainly from 1957 to 1979 [39,40,41,42,43,44,45,46]. At a minimum, serological evidence of *L. canicola* [40], *L. icterohaemorrhagiae,* and *L. serjoe* have been shown [41,46]. Considering that the slaughtered cattle in Gauteng, South Africa, originated from several provinces and that evidence of leptospirosis was shown in this study, it is necessary to investigate this same disease in other provinces and also to consider environmental contamination along the movement route of cattle and in value chain actors from other provinces to Gauteng Province [39,47].

Although serological evaluations typically return a higher prevalence for leptospirosis than isolation or PCR [28,39,48,49,50,51], we concluded that serological evaluations do not accurately estimate active infection or carrier status, and they may not demonstrate pathogen shedding [49,50,51]. Optimizing the isolation protocol for *Leptospira* spp. will provide increasing diagnostic efficiency for the pathogen using these more robust methods. In the current study, the *LipL32* gene region qPCR detected *Leptospira* DNA in 26.9% (50/186) of kidney tissues in slaughtered cattle, which was similar to reports in New Zealand (21.0%; 30/148) [39]. The qPCR determination of *Leptospira* DNA prevalence in New Zealand cattle, sheep, and pigs [39] is similar to the one from South Africa, which is perhaps a reflection of similarity in exposure factors for livestock and management practices.

Herr et al. [52] previously isolated *L. interrogans* serovar Pomona from cattle in South Africa. *L. canicola* was isolated from a dog and pigs by van Rensburg [53]. Gummow et al. [30] isolated *L. interrogans* serovar pomona from cattle urine and from an aborted fetus, as well as from pig kidney. In our study, *Leptospira interrogans* serovar Icterohaemorrhagiae and *L. borgpetersenii* serovar Hardjo bovis were determined from isolates and cattle kidneys using *SecY* region sequences, rather than serovar pomona as found in previous studies [30,52]. As determined in previous studies, risk factors for disease transmission in outbreaks included poor management of pig effluent and unhygienic drinking water [30]. In our study of the evaluated risk factors for the isolation of *Leptospira* spp., only sex (in cattle) was significant (*p* = 0.02). Infected livestock could be shedders of the pathogen capable of contaminating the environment (farms and abattoirs), exposing farmers, veterinarians, and abattoir workers to leptospirosis, thereby posing a zoonotic risk [38]. The possible effects of environmental factors such as rodent population, urine contamination, and sanitary practices on farms cannot be ignored [10,54].

In addition, although our study indicated a zero isolation rate in sheep in South Africa, it is not dismissive of the fact that sheep are susceptible. A study in Brazil reported a considerably higher frequency of isolation of *Leptospira* spp. from 46.2% (6/13) of sheep slaughtered in abattoirs [27,30]. *L. borgpetersenii* serovar Hardjo bovis identified in sheep and cattle using *secY* sequences in our study suggest circulation in the livestock population in the country. It is pertinent that, despite demonstrating the presence of *L. borgpetersenii* serovar Hardjo bovis, serologically and genetically, the central diagnostic laboratory at ARC-OVR uses an eight-antigen panel for MAT, excluding *L. borgpetersenii* serovar Hardjo bovis, which may suggest inadvertent under-reporting of leptospirosis in South Africa.

It is of potential clinical importance that the detection of the *LipL32* and *SecY* genes’ partial regions in this study indicates the presence of the pathogenic *Leptospira* virulence genes in the DNAs of the isolates and kidney tissues. Other researchers have used the detection of *LipL32* and the *SecY* genes’ partial region to determine the virulence of leptospires [15,16,21]. Since the prior clinical status of the livestock was not determined pre-slaughter in our study, it will be prudent to assess the clinical significance of the virulence gene-positive isolates of *Leptospira* spp. in future studies. This is because the possession of virulence genes by leptospires or other pathogens does not always lead to the expression of virulence in susceptible hosts [54,55,56]. Animal models, particularly hamsters, have been demonstrated to be very suitable for determining the virulence of *Leptospira* spp. [57,58].

## 5. Conclusions, Limitations, and Recommendations

The isolation of pathogenic *Leptospira* spp. at a rate of 3.9% (12/305) by bacteriological assay and the detection of pathogenic *Leptospira* DNA by PCR in 27.5% (84/305) of the kidneys of slaughtered livestock tested are indicative of the level of infection of livestock presented in the Gauteng provincial abattoirs and other provinces in the country. The data presented in this study contribute to a rich deposit of current data on the status of leptospirosis in Gauteng Province and South Africa at large, using bacteriological and molecular methods. This study presented the first documentation of molecular characterization studies on pathogenic *Leptospira* spp. in livestock in South Africa. Significantly, although sheep kidneys returned zero prevalence by culture for *Leptospira* spp., the same tissues yielded *Leptospira* DNA at a 42.2% detection rate (*n* = 19/45) by qPCR. We concluded that bacteriological assay alone will grossly underestimate the occurrence of *Leptospira* spp. in sheep.

Future research should consider increasing the sample size to provide ample and representative research information. The sensitization of the public regarding leptospirosis in livestock and humans, using validated risk communication and community engagement (RCCE) strategies, should be implemented in South Africa. In addition, improved technical know-how for diagnosing leptospirosis should be engendered through continuous capacity-building in the areas of bacteriology (culture), serology, and PCR. Finally, the inclusion of *L. borgpetersenii* serovar Hardjo bovis in the panel of antigens used to serotype the sera of animals for the occurrence of leptospirosis is recommended. It is imperative to conduct further studies of the isolation of *Leptospira* spp. from sheep in the country, using a larger sample size in abattoirs across the country to confirm the status of *Leptospira* infection.

## Data Availability

Data are available in a publicly accessible repository.

## Supplementary Materials

The following supporting information can be downloaded at: https://www.mdpi.com/article/10.3390/pathogens12050666/s1.
