# Supplementary material for: Molecular Characterization of Leptospira Species Detected in the Kidneys of Slaughtered Livestock in Abattoirs in Gauteng Province, South Africa
Source: Pathogens. 2023 Apr 30;12(5):666. doi: 10.3390/pathogens12050666 (PMC10223745; doi:10.3390/pathogens12050666)
Supplement: Supplementary file 1 [file pathogens-12-00666-s001.zip › pathogens-2355337-supplementary.pdf]

**Supplementary Data: Figures S1- S5**

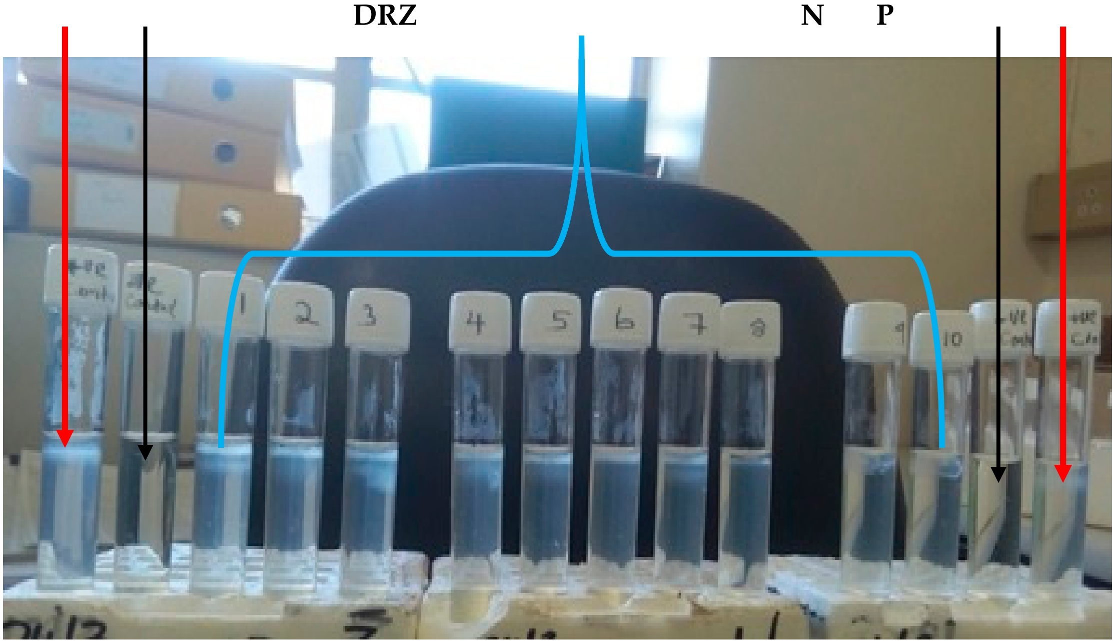


**Figure S1.** EMJH semi-solid medium inoculated with kidney tissues after 3 to 8 weeks incubated at 29 °C in this study. The positive controls (**P**) using *Leptospira* serovar, Grippotyphosa are indicated by red arrows, negative controls (**N**) using phosphate buffer saline (PBS) are indicated by the black arrows, and the Dinger's ring zone of leptospiral growth is indicated in the inoculated sample by the blue bracket. This morphological observation was further confirmed by observation under the dark field microscope for the active movement of leptospires. **Photo: By Dogonyaro, B.B. (graduate student Researcher), on the 24 March 2017.**

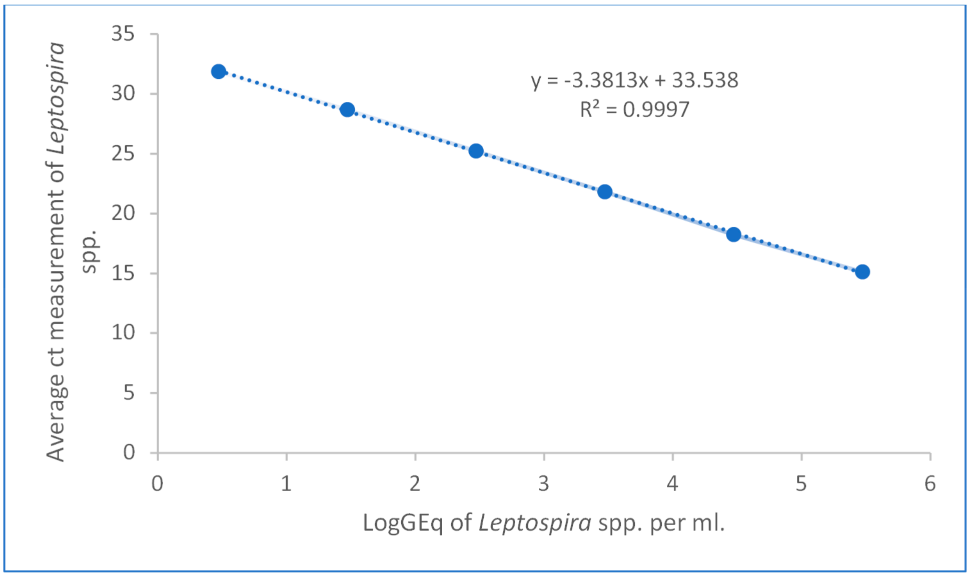


**Figure S2.** Standardized qPCR curve used to quantify the concentration of A standard stock positive control genomic DNA (*Leptospira interrogans,* serovar Copenhageni strain Fiocruz L1- 130) in GEq/ml targeting the *LipL32* gene region of the pathogenic *Leptospira* spp.

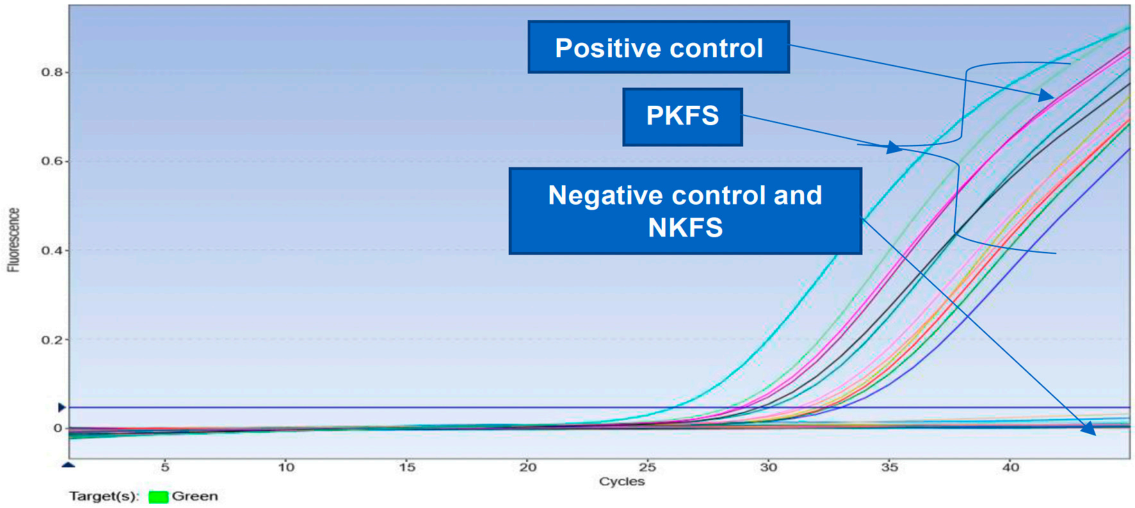


**Figure S3.** Fluorescence of hybridization of pathogenic *Leptospira* spp. Lipoprotein L32 gene (*LipL32*) probe using qPCR from cattle kidney samples, positive control (*Leptospira interrogans,* serovar Icterohaemorrhagiae strain), and negative control (ultra-pure water). PKFS=Positive kidney field samples; NKFS=Negative Kidney field samples.

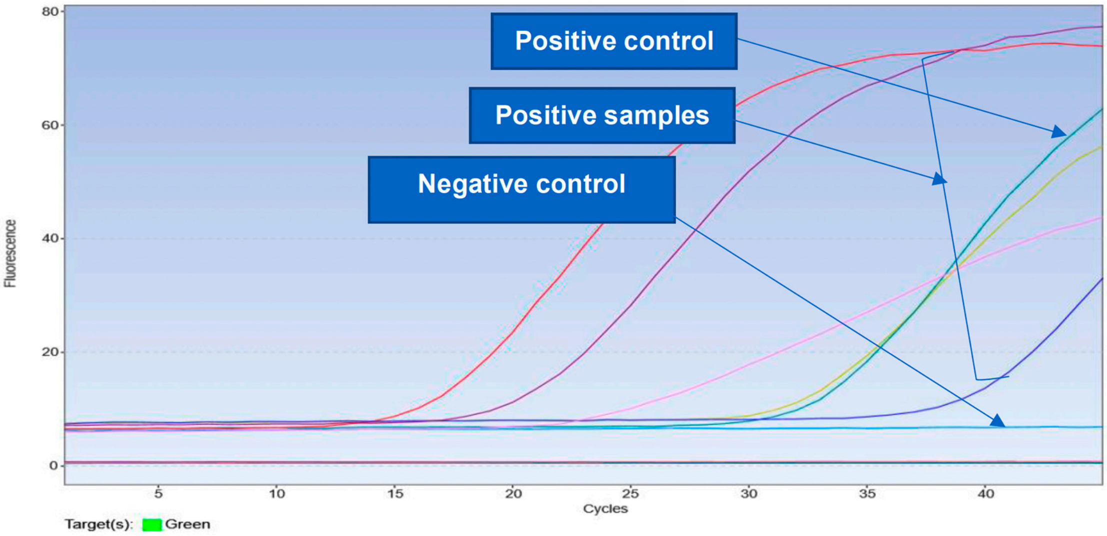


**Figure S4.** Fluorescence of hybridization Lipoprotein L32 gene (*LipL32*) probe of pathogenic *Leptospira* spp. using qPCR from pig kidneys, positive control (*Leptospira interrogans*, serovar Icterohaemorrhagiae strain, and negative control (ultra-pure water).

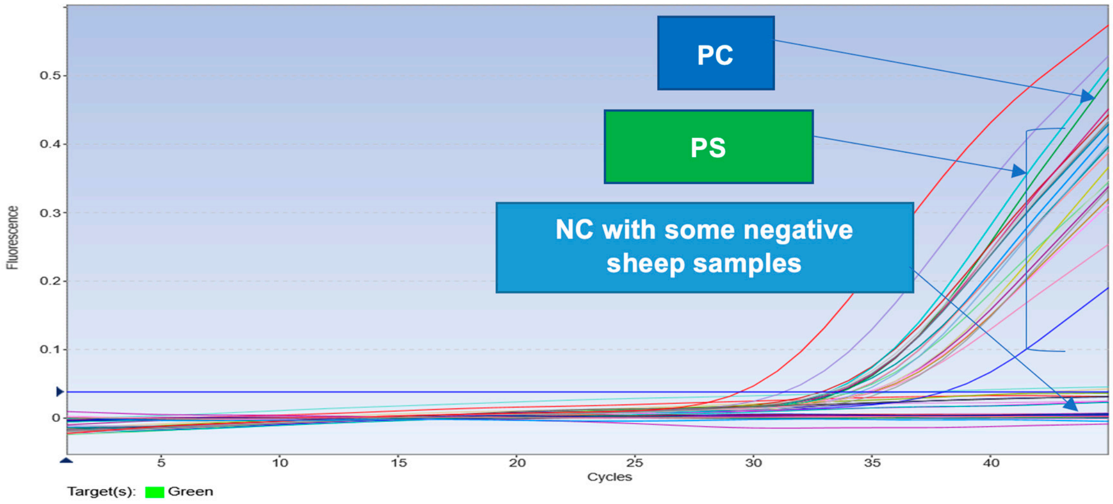


**Figure S5.** Fluorescence of hybridization Lipoprotein L32 gene (*LipL32*) probe of pathogenic *Leptospira* spp. using qPCR from sheep kidneys, positive control *Leptospira interrogans*, serovar Icterohaemorrhagiae strain (PC), and the negative control (NC) with other NKFS. PC= *Leptospira interrogans,* serovar icterohaemorrhagiae strain, Positive control; PS=Positive samples and NC=Negative control with some negative sheep samples.
